# Future Therapeutic Perspectives into the Alzheimer’s Disease Targeting the Oxidative Stress Hypothesis

**DOI:** 10.3390/molecules24234410

**Published:** 2019-12-03

**Authors:** Jéssika P. Teixeira, Alexandre A. de Castro, Flávia V. Soares, Elaine F. F. da Cunha, Teodorico C. Ramalho

**Affiliations:** 1 Department of Chemistry, Federal University of Lavras, 37200-000 Lavras, Minas Gerais, Brazil; jessikateixeira0904@gmail.com (J.P.T.); alexandre.a.castro@hotmail.com (A.A.d.C.); flaviavillela09@yahoo.com.br (F.V.S.); elaine_cunha@ufla.br (E.F.F.d.C.); 2 Center for Basic and Applied Research, Faculty of Informatics and Management, University of Hradec Kralove, 500 03 Hradec Kralove, Czech Republic

**Keywords:** Alzheimer’s disease, oxidative stress, antioxidants, free radicals, cellular respiration

## Abstract

Alzheimer’s disease (AD) is a neurodegenerative disease that is usually accompanied by aging, increasingly being the most common cause of dementia in the elderly. This disorder is characterized by the accumulation of beta amyloid plaques (Aβ) resulting from impaired amyloid precursor protein (APP) metabolism, together with the formation of neurofibrillary tangles and tau protein hyperphosphorylation. The exacerbated production of reactive oxygen species (ROS) triggers the process called oxidative stress, which increases neuronal cell abnormalities, most often followed by apoptosis, leading to cognitive dysfunction and dementia. In this context, the development of new therapies for the AD treatment is necessary. Antioxidants, for instance, are promising species for prevention and treatment because they are capable of disrupting the radical chain reaction, reducing the production of ROS. These species have also proven to be adjunctive to conventional treatments making them more effective. In this sense, several recently published works have focused their attention on oxidative stress and antioxidant species. Therefore, this review seeks to show the most relevant findings of these studies.

## 1. Introduction

Alzheimer’s disease (AD) is a gradual and neurodegenerative disorder, being the most common cause of dementia [1]. This pathogenicity currently affects more than 44 million people around the world. Regarding the modern health care system, the pathological condition that comes from the known neurodegenerative diseases (NDs) is one of the main health problems encountered nowadays. In this line, AD is one of the most challenging pathological frames for global scientists. AD generally comprises around 60–80% of all dementia events [2], being identified as the most predominant form of dementia among geriatric people [3]. It is important to mention that AD is characterized by progressive losses of neurons, brain functions and cognition functions [4].

Different kinds of risk factors like oxidative stress, obesity, diabetes, hypertension, air pollution, smoking, hyper cholesterolemia, among others, have a very significant role in the development of AD and in the development of its preventive measures. Physical exercise and nutritional factors have been shown as protective measures, contributing to its prevention [5]. It is noteworthy that there are some existing AD-related hypotheses, for instance, amyloid-β cascade hypothesis, tau protein hypothesis, cholinergic hypothesis, and oxidative hypothesis, which may help unveil the pathological factors behind the disease. In this review, the oxidative hypothesis is highlighted. In this line, the oxidative stress is also the commonality in the pathophysiology of NDs, such as AD, Parkinson’s disease (PD), Huntington’s disease (HD), amyotrophic lateral sclerosis (ALS), and multiple sclerosis (MS) [6].

In this approach, reactive oxygen species (ROS) are usually generated in the cell of living organisms. This is an outcome of normal cellular metabolism, being essential in the maintenance of cellular homeostasis. In normal conditions, low to moderate concentrations of ROS are important for diverse physiological activities, such as immune response, inflammation, among others [7]. On the other hand, the increased ROS production is harmful, leading to adverse oxidative modifications to cell components, such as the mitochondrial structures, which are important targets of ROS-induced damage [8]. In this context, this review presents the main roles of the oxidative stress in AD. The development of novel drugs and therapies taking into account this approach is also discussed.

## 2. Oxidative Stress

Reactive oxygen species (ROS) are important for the maintenance of hemostasis, as they function as a second messenger in the intracellular signaling cascade [9,10]. However, a redox imbalance or dysfunction of the antioxidant system, called oxidative stress (OS), can cause an excessive generation of ROS, which may lead to loss of function and cellular apoptosis [11].

The brain is an organ that requires a high oxygen demand to perform its activities properly, and, consequently, it is susceptible to the action of ROS. In addition, the brain is rich in polyunsaturated fatty acids that are exposed to peroxidation, presenting a high level of iron (a potent ROS catalyst), and it has a small amount of enzymes and other antioxidant substances [12]. Therefore, the oxidative stress plays an important role in the development of NDs such as AD. Most biomolecules can undergo oxidation, and in the case of AD, these biomolecules are mainly those from the neuronal membrane, which involves the oxidation of lipids, fatty acids, and proteins [13].

In this context, the oxidative stress plays a crucial role in the origin and development of AD, and altered levels of biomarkers may indicate AD. ROS are very unstable and reactive, presenting a very short half-life. In turn, the species they generate, after oxidizing biomolecules, are much more stable than themselves and can be used as biomarkers of oxidative damage. Examples of biomarkers include lipids, nucleic acids, and proteins in their oxidized states. Altered levels of antioxidant enzymes, such as catalases, superoxide dismutase, and glutathione peroxidase, as well as other antioxidant substances, can also be considered biomarkers [14]. For these facts, antioxidant species are promising in the prevention and treatment of oxidative stress-related diseases, which is the case for NDs. Lastly, it is important to mention that antioxidants are species that have the ability to sequester free radicals, thus, breaking the radical chain reaction and stabilizing both molecules [15].

### 2.1. Reactive Oxygen Species (ROS)

The ROS are highly reactive molecules because they have one or more unpaired valence shell electrons. These species can be free radicals derived from oxygen, such as superoxide anion (O_2_^.^) and hydroxyl radical (^.^OH), or non-radical molecules as it is the case of hydrogen peroxide (H_2_O_2_) [13]. In the brain, these species can be generated by exogenous sources, such as drugs wherein the mechanism of action is mediated by ROS, ionizing and UV radiation, environmental pollutants, quinone compounds, inflammatory cytokines, chemicals found in tobacco smoke, pharmaceuticals, and environmental toxins. However, there are also endogenous sources, where ROS production is mediated by mitochondrial and non-mitochondrial enzymes, including nicotinamide adenine dinucleotide phosphate—NADPH oxidase (NOX), xanthine oxidase (XO), granular endoplasmic reticulum cytochrome P450 and flavo-oxidase. The main source of endogenous generation is the respiratory chain and reduction–oxidation systems [16]. The superoxide radical, for example, is the most commonly found, and its main source of generation is the electron transport chain [17].

### 2.2. The Mitochondria and ROS Generation

Mitochondria are organelles that generate approximately 90% of their energy production in the form of ATP. In addition, they perform important functions such as fatty acid, lipid, amino acid, and nucleotide metabolism, cellular apoptosis, immune response, intracellular calcium ion homeostasis, intestinal microbiota maintenance and ROS production and protection. In this sense, mitochondrial dysfunction is observed in several NDs once the production of ATP generates superoxide as a byproduct, one of the main causes of oxidative stress [17].

**Scheme 1:**
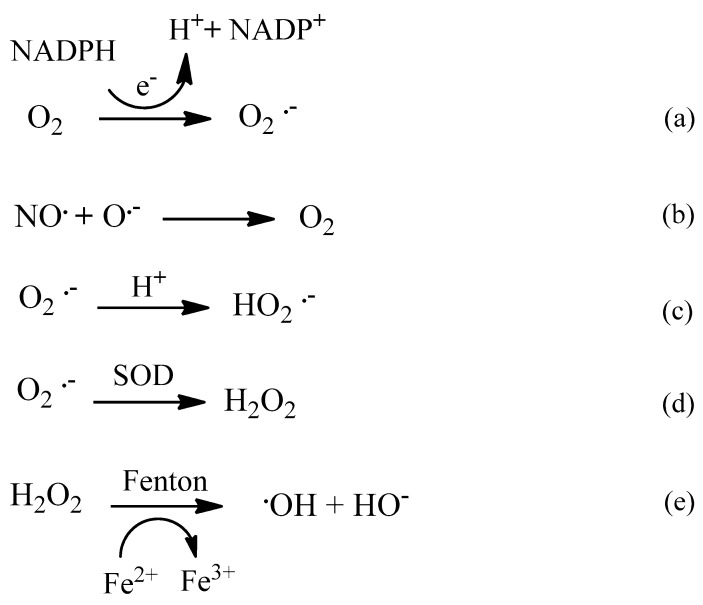
Endogenous generation reactions of ROS.

**Scheme 2:**
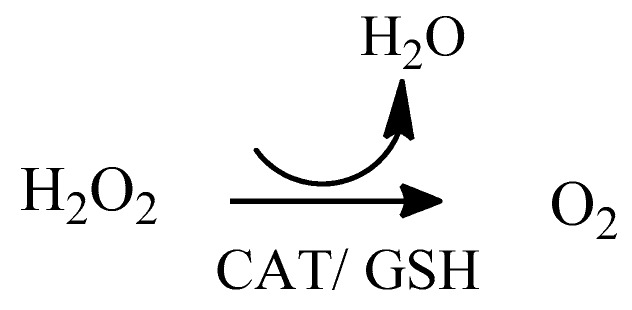
Action of antioxidant enzymes on ROS.

These organelles are the biggest consumers of intracellular oxygen and are, therefore, exposed to ROS. In the process of mitochondrial respiration, there is the formation of many reactive species such as hydrogen peroxide and superoxide anion. These radicals, in particular O_2_**^.^**, are formed through reduction reactions, because during the respiration process, in the electron transfer step, both electrons leak to oxygen [18,19]. The main production sites of the superoxide species are the respiratory-chain complexes I and III, but they can also be formed by NADPH oxidase (NOX), xanthine oxidase and cytochrome P450 reductase enzymatic activation (a). The superoxide radical can also react as a reactive nitrogen species (RNS), nitric acid, which is produced by nitric oxide synthase (NOS) during cell signaling, resulting in a powerful reactive species and significant contributor to oxidative stress called oxidizing peroxynitrite (ONOO–) (b) and can gain a hydrogen and form the species HO_2_^−^ (c) or it can be rapidly transformed into H_2_O_2_ by superoxide dismutase (SOD) (d). The hydrogen peroxide can be reduced into **^·^**OH by metal ions such as the iron through Fenton reaction (e) in Scheme 1. The hydroxyl radical is highly reactive and, therefore, undesirable to biomolecules. In order to prevent their formation, hydrogen peroxide is deactivated by antioxidant molecules such as glutathione peroxidase and catalase enzymes in Scheme 2 [16,20].

An abrupt increase in ROS stops the activity of the electron transport chain in mitochondria, resulting in the oxidation of molecules such as DNA and lipids [18]. In summary, NDs are characterized by the progressive death of neurons with causes often pointed to inflammatory processes and oxidative stress. AD, for example, is characterized by the accumulation of beta amyloid (Aβ) plaques resulting from an impairment of the metabolism of the amyloid precursor protein, together with the formation of neurofibrillary tangles from hyperphosphorylated tau protein. Increased Aβ production is directly related to neuron loss, tangle formation, synapse loss, and neurotransmission dysfunctions, and these dysfunctions are associated with NMDA glutamate receptor activation and oxidative stress [19].

The oxidative stress has been shown to induce constant changes in mitochondrial morphology, the mitochondrial networks that used to be tubular become reorganized into small (granular) spheres when there is prolonged and persistent oxidative damage, mitochondrial fragmentation and consequently apoptosis; this process reduces ATP production and results in an energy metabolism. Mitochondria are essential for the proper functioning of neurons, as synaptic transmission has a high energy demand. Under normal conditions, the dynamics of mitochondria allow them to travel through the axon and provide the energy needed for synapses at the endings of a neuron [17].

## 3. AD-Related Hypotheses

### 3.1. Amyloid-β Cascade Hypothesis

Alzheimer’s disease (AD) is a well-known pathology worldwide. This pathological frame is related to damages in neuronal functions, leading to progress loss of neurons and reducing brain volume. It is important to mention that the pathological mechanisms of AD are still unclear [21]. The most prevailing amyloid cascade hypothesis and sequential formation of neurofibrillary tangles (NFTs) are significant indicators of AD [22,23]. The neurotoxic Aβ is formed from the amyloid precursor protein (APP) due to aggregation processes of soluble oligomers, which result in the production of senile plaques (fibrils), being considered the main neuropathological marker of AD. APP is a substrate for two distinct enzymes: α- and β-secretases. These enzymes have an important role, being responsible for intersecting the extracellular domain of APP. The α- and β-secretase enzymes give rise to soluble *N*-terminal peptides (APPsα and APPsβ, respectively), as well as *C*-terminal fragments (CTFα and CTFβ), which are cleaved inside the membrane. This breaking process is driven by a third enzyme, the so-called γ-secretase [24]. Then, there is the release of p3 peptide from CTFα, or of Aβ from CTFβ. In this context, some important factors should be highlighted, e.g., the soluble peptide p3 presents no tendency to aggregate and form deposits (via non-amyloidogenic pathway). However, the processing of CTFβ by γ-secretase leads to the production of neurotoxic Aβ [25].

**Figure 1:**
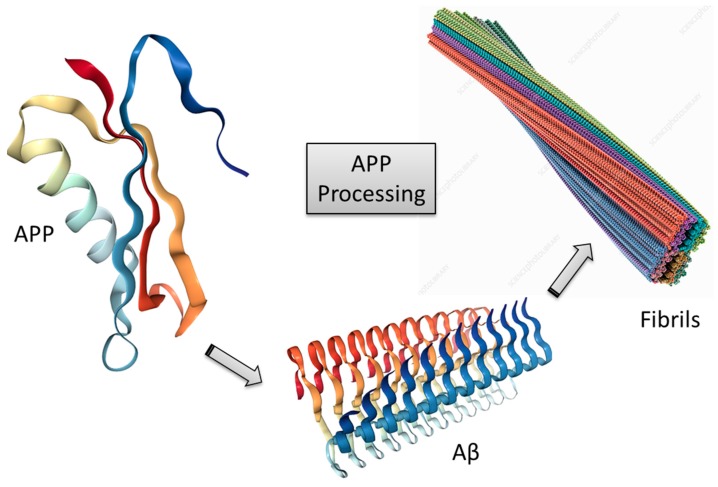
Amyloid precursor protein (APP) processing via amyloidogenic pathway.

In the past years to date, scientists around the world have discussed about the role of senile plaques in AD. Many studies have demonstrated that soluble oligomers have a more significant toxicity than fibrils. It is known that AD is related to the existence of Aβ oligomers and aggregates within the brain [24]. This standpoint is supported by indications that demonstrate that the concentration of senile plaques within the brain is not a good indication of the level of the disorder, and its reduction by using some therapeutic agent is not capable of reversing the disease progression [25,26]. Concerning the Aβ peptides, these species possess different lengths, ranging from 37 to 42 amino acids. On the other hand, it is well-known that the residue with 42 residues (Aβ_1–42_) has a characteristic pro-aggregating property. An important trend can be observed for most cells, whose main Aβ product is the less toxic isoform with 40 residues (Aβ_1–40_), accounting for over 50% of Aβ forms. The remaining species, with 37 to 39 residues, and the toxic Aβ_1–42_ account for only 5–20% of all Aβ forms. An abnormal augmentation in the production of Aβ_1–42_ increases the probability of developing AD [27]. In this context, many scientists search for novel and more efficient strategies for discovery of bioactive compounds capable of inhibiting the production and/or formation of Aβ deposits, by assisting in the removal of toxic Aβ or preventing its formation [25]. New therapies against Aβ should provide, for instance, reductions in the toxicity level of insoluble Aβ fibrils (Figure 1) [25,28].

Based on the evidence revealed so far, the amyloid hypothesis is still the most discussed AD-related theory, being a promising target for drug discovery [29,30,31,32,33]. According to this approach, there is the occurrence of events involving the proteolytic processing of APP [34]. The Aβ_1–42_ fragment is more likely to aggregate, being found in the core of neuritic plaques. Deposits of Aβ may occur anywhere that APP, β-secretase (BACE-1) and γ-secretase are found. As said previously, this is widely considered a key pathological component in AD [1,5,35].

Concerning the Aβ processing, we highlight once more that the fragments from α-secretase and γ-secretase cleavages, in this order, are demonstrated to present no toxicity [34,36,37,38,39]. The activation of α-secretase can favor the formation of non-amyloidogenic products from APP processing, thus being a remarkable drug target for decreasing pathogenic Aβ. Only a few compounds able to activate α-secretase have been verified [36,37,38]. Interestingly, evidences from some trials have shown that inhibitors of β- and γ-secretases could be effective in the remediation of early forms of AD. New studies suggest that the production of Aβ deposits takes place previous to the primary symptoms of the dementia [25,34]. Research lines concerning deposits and oligomers, along with the progressive phosphorylation of the tau protein, raise insights into the development and evolution of AD, bringing about advances in the discovery of novel remediation strategies [40].

### 3.2. Tau Protein Hypothesis

As mentioned previously, NFTs and helically twisted filaments of hyperphosphorylated tau are important pathogenetic modifications in AD. According to the β-amyloid hypothesis already discussed, NFT formation is preceded by deposits of Aβ [41]. The hyperphosphorylation of the tau protein results in the destabilization of the cytoskeleton and degeneration of nerve cells. In this line, it is worth mentioning that tau plays an important role in the stabilization of cytoskeletal microtubules. It is important to keep in mind that not only plaques formation leads to tau hyperphosphorylation, and this statement is supported by the fact that the neurofibrillary degeneration starts in the allocortex of the medial temporal lobe (entorhinal cortex and hippocampus), thus spreading to the associative isocortex. On the other hand, the entorhinal cortex is not susceptible to plaques formation [42]. Tau phosphorylation can take place at diverse unique sites of the protein and through multiple pathways. Note that the tau phosphorylation is critical to its function, but in the pathological frame, this process, which occurs by the action of kinase enzymes, is very harmful to the cell. The hyperphosphorylation of the tau protein cytoskeleton efficiently leads to the formation of tangles. This is an interesting biological target for drug development and discovery [43]. Concerning this hypothesis, novel therapies should involve the inhibition of tau phosphorylation, together with microtubule stabilization and prevention of tau oligomerization [25].

It is important to keep in mind that the hyperphosphorylated tau no longer binds to microtubules [44], leading to their destabilization. Increased levels of phosphorylated tau are important indicators of the disease, whose pathological process may result in neuronal injury and cell death [45]. Scientists have targeted tau protein for drug development in the sense of allowing for a reduction in the hyperphosphorylation process [46,47,48]. There are many kinases involved in tau hyperphosphorylation, for instance, glycogen synthase kinase (GSK)-3b and the cyclin-dependent kinase-5 (cdk5) are considerable active species in this pathological process. They can target multiple phosphorylation sites on tau [49]. Based on this hypothesis, researches are ongoing in order to explore different aspects of tau pathogenesis, such as modulation of tau gene expression, modulation of post-translational modifications, tau immunotherapy, and microtubule stabilization [50,51]. In addition, it should be highlighted that anti-tau immunotherapy might effectively become a promising strategy in AD treatment [51,52].

### 3.3. Cholinergic Hypothesis

**Figure 2:**
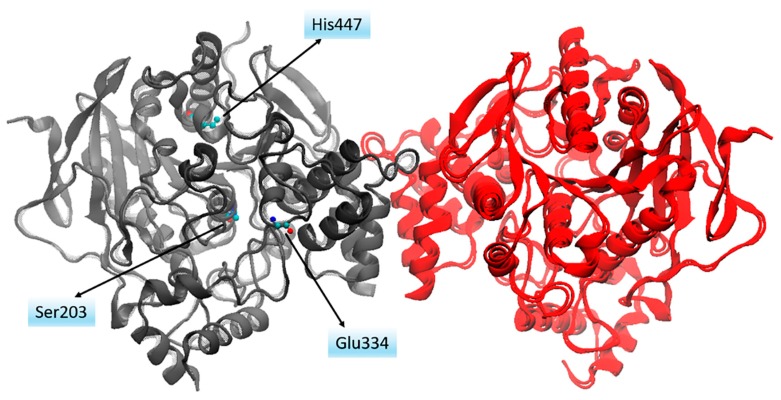
Representation of the three-dimensional structure of *Hss*AChE (PDB code = 5HF9), Reproduced from [58]. The catalytic residues are highlighted.

The cholinergic hypothesis is one of the major AD-related theories. Experimental investigations by employing the brain from AD patients as model demonstrate a reduced activity of some components in the cerebral cortex. In this context, it is possible to cite the activity related to choline acetyltransferase [53]. Interestingly, in vitro study indicates that Aβ peptide leads to the cholinergic neurotransmission inhibition [54,55]. Furthermore, other investigations demonstrated that the reduction in the level of nicotinic and muscarinic acetylcholine (ACh) receptor present in presynaptic cholinergic portions decreases cognitive function [56,57]. It should be highlighted that the acetylcholinesterase (AChE) enzyme (Figure 2) is the main therapeutic target within the cholinergic theory.

At present, the major therapies for AD treatment come from the cholinergic hypothesis [59,60]. It is important to highlight that experimental works by employing biopsy tissue and post-mortem brain tissues from AD patients revealed a decrease in the ACh neurotransmitter levels [57]. These remarkable findings suggest the importance of the discoveries of novel therapeutic approaches capable of reverting the degradation of cholinergic neurons and related loss of cholinergic neurotransmission [59,60,61]. Over the past years, efforts have been done in order to allow for an efficient AD treatment, e.g., with the development of cholinesterase inhibitors, choline precursors, among others [62]. Experimental investigations have shown significant symptomatic benefits through the administration of the cholinesterase inhibitors, bringing about improvements in cognitive, functional and behavioral symptoms in AD [63,64,65,66].

It is important to keep in mind that the failure in cholinergic neurotransmissions is an important factor concerning the characteristic learning and memory impairment observed in AD [67], and in this line, therapeutic strategies aiming at the cholinergic neurotransmission enhancement is the most important approach for treatment. Thus, there are few drugs approved by the Food and Drug Administration (FDA) to treat AD, being them the tacrine, donepezil, rivastigmine, and galantamine [68]. The cholinergic neurons are of great importance, composing the main neurotransmitter system involved in AD and cholinergic loss. They play important roles in maintaining the diverse functions and activities within the body, such as cortical activity, cerebral blood flow, learning, and activities-related memory [39]. According to this hypothesis, it is observed a significant decreased level of the ACh neurotransmitter [68]. The cholinesterase inhibitors act by inhibiting and reducing the breakage of ACh, thus enhancing the cholinergic neurotransmission. Among the AD-related hypotheses presented here, we highlight in this review the oxidative hypothesis, which involves important aspects related to the disorder, being an important target for drug discovery and modifying therapies.

## 4. Antioxidants

The body is composed of an antioxidant protection system, which has the function of protecting healthy cells against free radicals. These radicals are formed in normal cellular metabolism and in pathological conditions. Although fundamental to health, when in excess they can promote the oxidation of biological molecules, triggering what is called oxidative stress. The importance of antioxidants lies in the fact that they are able to regulate the amount of these radicals in the body [69].

These free radicals whose unpaired electron is centered on oxygen or nitrogen atoms are then referred to as ROS (reactive oxygen species) and RNS (reactive nitrogen species), as commented previously. They are involved in several processes, including energy production, immunity and cellular defense. However, when in excess they present detrimental effects, such as membrane lipid peroxidation and aggression to tissue and membrane proteins, enzymes, carbohydrates, and DNA [70,71,72]. In this context, the cell membrane is one of the cell components most affected by the action of ROS, due to lipid peroxidation, which causes changes in membrane structure and permeability [73].

Thus, each cell that uses oxygen and enzymes to perform its functions will be subject to free radical reactions, with a high chance of causing damage to the cell and the body in general. In this line, antioxidants help reduce the oxidative stress situation (imbalance between free radical and antioxidant levels), since they are molecules capable of donating an electron to free radicals preventing cellular destabilization [74].

**Scheme 3:**
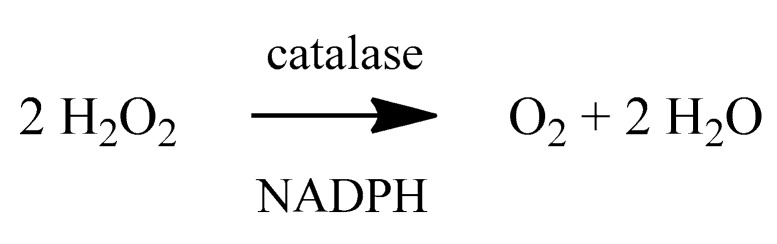
Dismutation of hydrogen peroxide into oxygen and water.

Three antioxidant enzyme systems are known. The first one consists of two types of SOD enzymes, which catalyze the dismutation of the superoxide anion radical O_2_^−^, converting it into oxygen and hydrogen peroxide [75]. The second antioxidant system is considered simpler, being formed by the catalase enzyme that acts in the dismutation of the hydrogen peroxide into oxygen and water, as shown in Scheme 3.

**Scheme 4:**
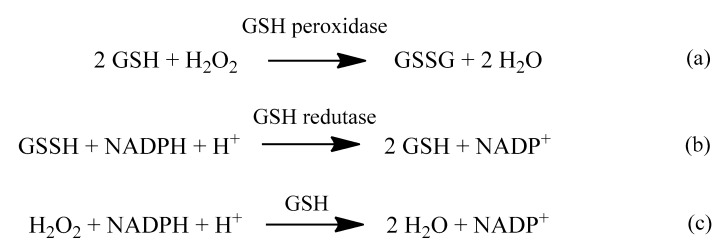
Equations of the reduction of H_2_O_2_ into H_2_O by GSH in the presence of GPx, with disulfide bridge formation and then GSH regeneration.

The third system consists of glutathione (GSH) together with two enzymes, glutathione peroxidase (GPx or GSH-Px) and glutathione reductase (GR or GSH-Rd), and the presence of selenium in the enzyme (selene–cysteine) denotes the importance of this metal and its role as an antioxidant within the body. This system also catalyzes the dismutation of hydrogen peroxide into water and oxygen, and glutathione operates in cycles between its oxidized and reduced form [70], as shown in Scheme 4. 

Some studies point to oxidative reactions as an important factor in pathological processes [76], including NDs such as AD [77]. Over the last decades, many hypotheses have been generated in order to explain this pathophysiology, as presented previously [21,77,78,79]. To date, it is known that oxidative stress plays a central role in AD, although it is not clear whether this is a disease triggering event or it is a side effect [80].

In this context, antioxidants can act in two ways under free radicals: Inhibiting their formation and repairing the damage already caused. The first one is related to the inhibition of chain reactions involving their formation; and the second one, in the removal of damaged cells, followed by reconstitution of cell membranes. In this sense, it is important to mention the two antioxidant self-defense systems that the human body presents: The enzymatic (endogenous) and non-enzymatic (exogenous) systems. The non-enzymatic is made up of groups of substances such as vitamins, plant substances and minerals that can be ingested through the diet. In turn, the enzymatic system is formed by a set of enzymes naturally produced by the body [73,81].

The endogenous self-defense system, however, tends to be reduced with the natural aging process, as the production of antioxidant enzymes loses its efficiency over the years. Therefore, it is important to maintain the quality of the non-enzymatic defense system by eating antioxidant-rich foods. With respect to this exogenous system, the antioxidant potential in vivo depends on some variables, for instance, absorption and bioavailability under physiological conditions, ideal plasma concentration, types of free radicals generated in the oxidative process, along with the cell compartment where they were generated and how they were generated [81].

The use of vitamin-rich diets is an important adjunct in the treatment of NDs. Since, as already discussed, vitamins are powerful antioxidants that directly influence the fight against free radicals, reducing oxidative stress, inflammatory processes and loss of neurons [82].

Vitamin A plays a major role in the development of neurons and remains present in the nervous system throughout life. It also plays a role with beta-carotene in protecting and regenerating neurons during the process of neurodegeneration, inhibiting formation and aggregation of Aβ plaques. It may also prevent impaired cognition in AD and improve memory performance and spatial learning. Studies have shown low levels of vitamin A and beta carotenes in AD patients, compared to healthy people [82,83].

B-complex vitamins such as B6 (pyridoxine), B9 (folic acid), and B12 (cobalamin) have been shown to lower the level of hemocysteine, a compound responsible for increased neuron toxicity and brain gray matter atrophy. Inappropriate supply of these vitamins is related to shrinkage of the cerebral cortex, cognitive impairment and dementia, and increased calcium in cells leading to apoptosis and accumulation of Aβ peptides. Plasma levels of these vitamins in AD patients were also considered deficient [82].

The use of high doses of B-complex vitamin has been shown to decrease gray matter atrophy caused by hemocysteine, and in particular B12, it has been shown to be an excellent adjunct in the treatment of cognitive decline associated with age or NDs [84].

Vitamin K is also linked to the development of AD, and it is believed that its protection is related to the reduction of oxidative stress, because it can prevent cell death after activation of the lipoxygenase pathway. Vitamin D levels are also low in patients with AD, and when administered together with a famous AD drug, the memantine, it was more effective than when the drug was administered alone [85].

Given the known antioxidants, the most outstanding micromolecules are ascorbic acid (vitamin C) and α-tocopherol (vitamin E) [75,86]. Vitamin C is commonly found in the human body in the form of ascorbate, located in organic tissues. It plays fundamental metabolic roles, acting as a reducing agent, by reducing transition metals (Fe^3+^ and Cu^2+^) present in the enzymes active site or in free forms in the body. Due to the fact that it is a good reducing agent, ascorbate can be oxidized by most ROS and RNS that arrive or are formed in aqueous compartments of organic tissues [70]. It can also prevent aggregation of Aβ peptides. Vitamin C supplementation improves SOD levels, which helps to reduce oxidative stress and the damage it causes. Research shows that normal vitamin C intake has neuroprotective effects and may help improve cognition in AD patients [86].

On the other hand, among all known tocopherols, the vitamin E has been considered the most biologically active, being the main fat-soluble antioxidant in cell membranes. The fact of being fat-soluble confers the property of accumulating inside the membranes and being transported by lipoproteins, especially low-density lipoproteins (LDL). It is stored in various tissues, especially in the liver, adipose tissue and muscle, and its excretion may take place through feces, biliary tract, or skin [87]. Vitamin E is primarily responsible for the removal of free radicals in the erythrocyte membrane and has an important role in stopping the spread of lipoperoxidation, thus acting to prevent hemolysis by maintaining membrane stability. Deficiency of this vitamin can lead to neuron destruction and brain atrophy. Vitamin E has the power to inhibit neuronal death caused by inflammatory processes [69,86].

Important advances related to the use of antioxidants and AD have gained space in therapeutic researches [88,89]. Some observational studies have suggested that antioxidant supplementation, including vitamins C and E, or a diet rich in these nutrients may prevent oxidative stress, thus lowering the risk for the disorder [90].

Medicinal plants are promising sources of treatment for various diseases. This is an ancient practice that gave rise to synthetic drugs of paramount importance used nowadays. In traditional medicine, several plants have been indicated for the treatment of NDs. Among these plants, it is possible to mention Asian sparkle, Aloe arborescens, Proanthocyanidin, Capparis spinosa L, Alpinia galanga L, and Abelmoschus esculentus (okra) that have been shown to reduce oxidative stress in AD, demonstrating a neuroprotective effect of brain aging caused by D-galactose in mice. Plants with rutin in their composition (vitamin P) have shown a protective role against neurocytotoxicity. Phenolic compounds present in plants are also pointed as potent promising antioxidant agents in the treatment and prevention of AD [91].

*Turmeric* is a plant of Asian origin, from the Zingiberaceae family, that has in its composition curcumininoids, especially and mostly curcumin (77%). In vitro studies have shown that this compound can bind to Aβ peptides, preventing their aggregation and the formation of characteristic AD plaques. It can also act as an antioxidant anti-inflammatory agent and has been shown to be effective in inducing microglia activation in mice [92,93].

Regarding oxidative stress, it is highlighted the disturbed metabolism of redox active metals (Fe, Cu) and redox inactive metals (Zn). The brain is a specialized organ that normally concentrates Fe, Cu, and Zn in the neocortex. There are strong indications that the homeostasis of these metals is significantly altered in AD brains. Studies show that these metals accumulate in the neuropil of the AD brain. It is worth mentioning that the redox active Fe and Cu are implicated in free radical reactions. Redox active metals catalyze the formation of these free radicals by a variety of reactions, in addition to Fenton reaction that is of key significance. These species are concentrated in the regions of the brain most affected by the disorder. Interestingly, the redox active Fe is found within amyloid deposits in the human brain, as well as in the neocortex of mice models of AD. A therapeutic approach would consist in the use of small molecules (metal chelators) to deplete the deposits of excess Cu, Zn, and Fe [94].

Small oligomers of Aβ and tau have also been receiving increased attention due to their significant correlation with neurotoxicity. Oxidative stress could be involved in the clearance of Aβ. The oxidation of biomolecules in the context of AD is mostly related to neuronal membrane biomolecules and to a disruption of membrane integrity. This involves the oxidation of lipids, proteins and nucleic acids, and impairment of Aβ clearance due to the low density lipoprotein receptor-related protein (LRP1) oxidation. There are indications that Aβ would oxidize LRP1, leading to accumulation of the neurotoxic Aβ in the brain. LRP1 is a protein responsible for the efflux of Aβ from the brain to the blood, across the blood–brain barrier (BBB). Nevertheless, the LRP1 activity is decreased in AD [95]. In this context, Aβ, by oxidizing LRP1, can potentially lead to disruption of its own clearance, resulting in an increased accumulation of Aβ in the brain, which is one of the determinant factors in AD. The tau protein also constitutes a target for oxidative stress in AD. For purposes of exemplification, we can cite 4-HNE (4-HydroxyNonenal) that is capable of inducing modifications of tau protein conformation, supporting the involvement of oxidative stress (remarkably induced by Aβ) in the pathogenesis of AD, by favoring the NFTs formation [11,96].

As discussed previously, the ROS production is increased within the mitochondria under some conditions of stress, in addition to aging. This fact, along with the absence of an effective antioxidant system, very significantly increases the probability of developing AD. It is demonstrated that the brain of AD patients shows a significant extension of oxidative impairment. Note that ROS have an important role in activating BACE-1 and γ-secretase enzymes, leading to increased Aβ formation and abnormal agglomeration of Aβ fibrils in the brain of AD patients. It is important to notice that Aβ and APP may themselves also prompt the formation of ROS. One well-known enzyme, called monoamine oxidase (MAO), is also indicated to be involved in AD due to the increased synthesis of ROS [97].

## 5. Other Relevant Therapeutic Approaches in AD Treatment

Many clinical trials in AD patients have investigated the therapeutic potential of BACE-1 inhibition. Unfortunately, these trials have failed to show a significant reduction in cognitive decline when administered to patients in a more advanced stage of the disorder. Studies suggest that this treatment was ineffective because of late administration in the disease course. The scientists employed two-photon microscopy in live mice in order to evaluate the kinetics of Aβ plaques deposition in different situations, before and during BACE-1 inhibitor treatment. The inhibition of this enzyme potentially blocked novel Aβ plaques generation, but it was less efficient in slowing the growth of existing plaques. In this context, it was concluded that the optimal timing for treatment would be in an early stage of the illness, prior to the widespread Aβ plaques formation [98].

Besides BACE-1, γ-secretase has also been considered as a therapeutic target for some diseases, such as AD. Potent γ-secretase inhibitors (GSIs) have been developed and tested in patients with AD and cancer. However, the clinical development of GSIs requires careful evaluation of the benefits and risks involved. Along with GSIs, other agents denominated γ-secretase modulators (GSMs) are still in development as AD therapeutics. These compounds do not inhibit γ-secretase, but by modulating it, the profile of the secreted Aβ peptides shifts. The work from Golde et al. (2013) describes the current state of development of these GSIs and GSMs agents [99].

Novel therapeutic approaches aim at the reduction of the generation of deleterious tau aggregation intermediates and tau filaments. The hyperphosphorylation process of the tau protein could be prevented by employing protein kinase inhibitors. In the work by Holzer et al. (2018), it was identified inhibitors of three kinases with relevance to tau pathology. The authors were able to demonstrate a reduction in tau protein phosphorylation. New inhibitors of three protein kinases (gsk-3β, cdk5, and cdk1) were developed, taking into account their activity in relation to tau phosphorylation, as well as the stage of nucleation or aggregation (tau–tau interaction) in cells. All inhibitors showed strong effects toward the kinases investigated, with highlights on gsk-3β in nanomolar ranges [100]. Note that the tau protein has become an increasingly important target in drug discovery, contributing to the development of more efficient therapies for the AD treatment [101,102].

Taking into account the current strategies for combating AD, their development is based on a multifunctional approach (multitarget-directed ligands), such as Aβ antiaggregation, antioxidant, and metal-chelating properties, together with AChE inhibitory activity. In this line, the employment of flavonoids has shown important therapeutic activities. According to publication from Simunkova et al. (2019) [103], many flavonoids, in addition to some alkaloids and coumarin compounds, exhibited therapeutic benefits for the AD treatment, e.g., with antioxidant, metal-chelating and inhibitory activities toward AChE. Active molecules, such as resveratrol, trolox and coumarin are capable of suppressing ROS formation, by means of chelating metals and decreasing their catalytic activity, with the possibility of directly scavenging ROS. It is also important to mention the employment of herbal drugs, which can potentially cross the BBB and exert activity through the AChE inhibition, reduction of neuroinflammation and Aβ aggregates, and redox active metals chelation. Some examples of herbal drugs are huperzine A and B, corilagen, among others. These therapeutic approaches are of special importance in AD treatment, coming to the light a range of possibilities, capable of targeting different pathological targets related to the disorder. For instance, the flavonoids, besides acting as antioxidants, are signaling molecules, being able to enhance cognition and slow down the disease progression. In this regard, we must cite one important molecule, the well-known quercetin, which has the potential to protect neurons from oxidative stress [103].

As shown in this review, it is important to notice that remarkable researches point to oxidative stress as a factor associated with the early development of AD, which supports the search for novel efficient therapeutic approaches to combat neuronal oxidative damages. Based on the information presented in this review, note that there are relevant possibilities that could be essential in drug discovery for AD treatment and prevention. Faced with this worrying world scenario, the research projects in this area are turning out to be crucial in order to find novel forms of treatment and therapies. The increasing number of new cases of dementia annually shows the importance of these studies. Theoretical investigations can potentially contribute to the discovery of new drugs and therapies. Diverse computational tools have been successfully employed with the study of biomacromolecules, approaching different infirmities and bringing about remarkable advances in medicinal chemistry [104,105,106,107,108,109].

## 6. Conclusions

Alzheimer’s disease (AD) is a type of pathology of global concern, which has become a serious public health problem. Several researchers worldwide have strived in the search for novel forms of AD treatment and prevention. However, to date, there is no therapy able to induce reversal of the illness. The drugs currently used only bring about symptomatic benefits, but without reversing the pathological process. In this line, the discovery of new therapies is undoubtedly an important goal, in order to provide better and more efficient treatment conditions for AD patients. As discussed along this review, note that the oxidative stress is an important pathological factor in AD, being a substantial therapeutic target for the development of remediation methods. In this context, the antioxidants are remarkable contributors for these therapeutic purposes, along with the methods for combating Aβ and tau aggregation.
